# “No One is Going to Listen to a Bunch of Kids”: Youth Requests for Radical Listening and Epistemic Justice

**DOI:** 10.1177/0044118X251381839

**Published:** 2025-10-09

**Authors:** S. Sylvane Vaccarino-Ruiz, Alisha Solomon, Regina D. Langhout

**Affiliations:** 1 University of California, Santa Cruz, USA

**Keywords:** civic engagement, discrimination, focus groups, role models/mentors, prosocial involvement, social inequality, alienation

## Abstract

Many of the challenges youth in youth-adult collaborations face are rooted in adultism, which impedes efforts toward justice. Research suggests that rather than merely focusing on inclusion, adults need to meaningfully support youth, since youth report not being taken seriously by adults. Radical listening theory offers relational, epistemic, and embodied practices. Yet, unsurprisingly, youth perspectives still need to be seriously understood and affirmed regarding their desires for partnership and listening. Survey and focus groups analyses with youth and adults in the county suggest that mutual respect, a nuanced form of mentorship, and being validated as epistemic partners is vital.

Participation in communal discourse and activism is restricted and facilitated along social categories and power dynamics, including age. In the U.S., social constructions of youth often restrict their legitimacy and inclusion in communal matters, which is a form of epistemic injustice known as testimonial injustice (Fricker, 2007; Glass & Newman, 2015). Yet, young people, often youth of color, frequently lead social change (Ginwright, 2015). Adultism, the age-based system of oppression characterized by adult-centric policies, practices, and paternalistic relations subjugating youth (LeFrançois, 2014), obstructs youth’s meaningful inclusion into community change. Adultist notions of youth as incompetent and unreliable narrators, thus illegitimate knowledge producers, shapes how adults engage with youth. Consequently, a steady call urges adult youth-workers to disrupt adultism and work toward epistemic justice (Debower, et al., 2023; Ginwright, 2015; Liou & Literat, 2020; Vaccarino-Ruiz et al., 2021; Watts & Flanagan, 2007).

Youth-adult collaboration models generally demonstrate common values: resist tokenizing youth, integrate youth ideas, create spaces for youth (and adults) to think critically and dialogically, balance power and decision making between youth and adults, increase youth access to social networks, and hear, respect, and take seriously youth opinions (L. Camino, 2005; Corney et al, 2022; Hall, 2020; Jones & Perkins, 2006; Zeldin et al., 2013). Similarly, the youth adult partnership literature suggests relational aspects and co-construction of knowledge and authority are important for sustainability (Nalani, et al., 2021). These facets begin to disrupt adultism in partnerships, because oppression is often maintained by social division and unjust assumptions about knowledge (Moane, 2011). Although adults and youth need to address systems (e.g., policy, structures), this article focuses on an interpersonal practice adults can embody with youth to practice solidarity and accountability in service of epistemic justice: listening.

Radical listening affirms the voices and knowledge of differently positioned groups (Agnello, 2016). Radical listening is distinctive because it humanizes the speaker (Heldke, 2007), acknowledges and attends to the distracting “noise” shaped by power dynamics (Kress & Frazier-Booth, 2016), and seeks to understand the epistemic standpoint of the speaker (Tobin, 2009). Indeed, listening is implicated in power’s asymmetry, which subjugates oppressed groups’ knowledges. Radical listening can facilitate relational bonds, which aligns with the adult solidarity with youth goal. Yet, a deeper understanding of how radical listening happens and how youth desire listening is missing from the literature.

We aim to gain insight into youth desires regarding adult collaboration, focusing on how youth want adults to listen, to challenge adultism. We inquire how the radical listening framework compares with our data, and how it complements literature on youth-adult collaboration.

## Youth-Adult Collaboration

For adults collaborating with youth, it is insufficient to plug youth into adult spaces and assume their participation will organically flow or not be subject to extractivism (Hall, 2020; Jones & Perkins, 2006; V. Morgan, 2020; Percy-Smith & Thomas, 2023). Accordingly, various youth-adult collaboration models signal how youth and adults can collaborate. For example, a program might follow: (1) youth participatory action research, where youth collaborate with adults to research social issues, and create and implement social actions, (2) youth organizing, where youth train in community organizing and advocacy to create social change, or (3) youth-adult partnerships, where youth and adults collaborate long-term to improve youth programs or address community issues (Hall, 2020).

In each model, youth experience is important given the U.S. is age segregated and most societies are adultist, which shapes youth-adult collaboration and assumptions about knowledge production, and is named as the biggest barrier to youth participation (Augsberger et al., 2017; Rogoff, 2003). Therefore, we need critical inquiry on *how* youth experience adult collaboration, and how they want adults to listen (Corney et al., 2022; Jones & Perkins, 2006; Liou & Literat, 2020; Taft, 2015; Zeldin et al., 2013). Indeed, how youth experience adult-youth interactions is understudied (Gordon, 2007; Griffith et al., 2018; Liou & Literat, 2020), and under-theorized (Nalani et al., 2021). When examined, even in contexts with decades-long child/youth-centered movements, young people still critique adultism (Taft, 2015). Moreover, adults working with youth often name needing to show restraint in their sharing and guidance (Larson et al., 2016), yet sometimes overcorrect and offer next to no guidance (L. Camino, 2005). Perhaps these situations are why researchers call for better understanding the depth and breadth of youth participation (Zeldin et al., 2017), experiences across sectors (London & McLaughlin, 2014; To et al., 2021), and how adults can collaborate with youth (Liou & Literat, 2020; Richards-Schuster & Timmermans, 2017).

Critical inquiry on sharing power is important given many youth-adult collaboration models are created by adults, either building from other adult-based participation and/or empowerment models, or by observing youth-adult collaborations (L. Camino, 2005; Griffith et al., 2018; Hart, 1992; Wong et al. 2010), rather than talking with youth, or youth reflecting on power dynamics (To et al., 2021). Partly for these reasons, the models and youth-adult collaboration practices are critiqued for their adult-centering conceptualization of participation (Augsberger et al., 2017; Wong et al., 2010; Zeldin et al., 2007).

To contend with adultism in youth-adult collaborations, we must consider socio-political factors. Indeed, young people’s participation with institutions varies based on the intersections of social dimensions like race, social class, citizenship status, age, and more (Augsberger et al., 2017; Braithwaite & Deane, 2023; Deane et al, 2025; Gordon, 2007; Jones & Perkins, 2006). Youth with less social power are often taken less seriously by adults. Because of deficit-based narratives about youth of color and working class/poor youth, youth-adult collaboration focusing on youth strengths and capacities may be important in facilitating a more strengths-based and supportive counter-narrative about youth and their capacities (Zeldin et al., 2017).

Despite narrow conceptualizations of youths’ capacities as change agents in the U.S., youth play active roles in political engagement and movement building around the globe. For example, child activists within Child Parliaments in Bangladesh and Ghana successfully stopped child marriages through their long-term investment and social networks (Cuevas-Parra & Tisdall, 2022). In the Peruvian movement of working children, people are decision makers and organizers of campaigns for children in schools, families, and workplaces (Taft, 2015). Māori and Pacific Island youth in Aotearoa (New Zealand) are at the forefront of the climate change movement, linking climate crisis with settler colonialism (Ritchie, 2020). Palestinian youth practice political agency by promoting democracy among Palestinian factions and institutions while resisting Israeli occupation and colonial violence through boycotting, protesting, and building global solidarity networks (Habashi, 2017; Mokadi & Yousef, 2024). Global examples demonstrate youth activism is sophisticated, critical, and methodologically diverse. Yet these examples cite intergenerational collaboration as an enduring struggle. Adults, especially in the U.S., have much to learn from youth regarding collaboration.

Few studies ask youth about their experiences with adults in school or out-of-school settings, or in an organizing space. When asked, youth discuss the importance of mutual respect and care; non-tokenized project responsibilities or meaningful roles; co-creating safe and affirming spaces; being seen as a whole person; and being trusted, listened to, and affirmed (L. A. Camino, 2000; Deane et al, 2025; Gordon, 2007; Griffith et al., 2018; Liou & Literat, 2020; Mitra, 2009; Nalani et al., 2021; Taft, 2015). Youth wanted adults to: avoid patronizing admiration of youth achievements, have a systems-level understanding of adultism, and create room for youth-led spaces (Gordon & Taft, 2011; Gordon, 2007). They also wanted adults to guide without taking over, regularly ask youth how to support them, educate themselves on relevant social issues, share resources with youth, and be encouraging and vulnerable (L. A. Camino, 2000; Gordon, 2007; Griffith et al., 2018; Liou & Literat, 2020; Mitra, 2009; Nalani et al., 2021; Taft, 2015).

Similarly, one review of youth-adult collaboration studies theorized a multi-level framework for sustainability within youth and adult programs; they suggest to address relational challenges leaving youth feeling silenced, adults need training in not replicating unjust norms when working with youth (Nalani et al., 2021). Two goals are relevant: (1) Community building activities where youth and adults teach each other something new can build mutual respect, trust, and openness (Nalani et al., 2021; Zeller-Berkman et al., 2020) and (2) co-constructing knowledge and authority can facilitate long-term youth participation (Mitra, 2008; Nalani et al., 2021). Youth and adult researchers agree, community building and co-construction of knowledge are important.

Specific contexts may be more likely to facilitate more equitable power sharing among youth and adults. These contexts appear to include participatory design, with adults trained on adultism (Deane et al, 2025), youth decision making with adult guidance in a post-Katrina disaster relief collaboration (Osofsky et al., 2018), and meaningful inclusion of youth with diverse experiences as authentic decision makers around mental health/addiction research and programming (Heffernan et al., 2017). Although these contexts vary, adults centering youth as meaningful social actors is consistent.

The literature suggests relational bonds and power sharing are important, yet specific ways bonds are built has not been substantiated by youth. Especially in work aiming for social justice, how practices are embodied, socio-political, and compared across sectors is unclear. A praxis framework prioritizing social transformation and solidarity between teachers and students can be useful. Specifically, a critical pedagogy framework should offer practices to support relationality and challenge adultism. Focusing on adult listening can hold important insights into partnership, accomplice-ship, and solidarity with young agents of social change. Not any listening will do, as underlying adultist assumptions about knowledge production and legitimacy are likely to block the accomplice-ship needed. Radical listening may offer a model.

## Radical Listening

Critical pedagogy scholars draw from Kincheloe’s radical love construct to theorize four radical listening dimensions: relational commitments, critical dialogue, epistemic justice, and attention to the socio-political fabric (Agnello, 2016). Radical love qualities, like dedication to connecting and developing humanizing interrelations to work together toward transformation, lead to radical listening. Thus, relational commitments are important to radical listening. “Radical listening involves consciously valuing others by attempting to hear what the speaker is saying for the [intended] meaning. . . rather than the meaning the listener interprets through his/her own view of the world” (Winchell et al., 2016, p. 101).

Radical listening is at the heart of dialogue where participants remain open to changing themselves by listening to others (Kress & Krueger-Henney, 2016; Moore, 2018). Radical listeners give attention to common barriers to dialogic processes for social transformation like imposing meaning and judgment, binary thinking, and inequitable power dynamics (Moore, 2018). For inquiry-based, action-oriented, and justice seeking dialogue to occur, parties must intentionally and actively listen with humility.

Radical listeners have a deep desire to understand another’s philosophical standpoints and value-based positions (Moore, 2018; Tobin, 2009) without trying to change the speaker. Listening is based on countering epistemologies seeking universal truth (Winchell et al., 2016). Radical listening is actively political because it resists neoliberal and colonial ways of knowing and rejects narratives dehumanizing subaltern groups (Kress, 2016). Radical listening is distinct because of its attention to the epistemological aspects of power, yet epistemic justice has not been explicitly defined in this framework. Staying open to other knowledges and the dialogic process of knowledge construction requires attention to power embedded in different locations and speaker positionality. Interrogation of power involves consideration of testimonial injustice, which occurs when perspectives of marginalized group members are seen as credible due to their positionality (Fricker, 2007). Testimonial justice is distinct from hermeneutical injustice, which arises from a gap in the epistemic resources or frameworks needed to understand disempowered parties’ experiences (Fricker, 2007).

Radical listening is distinctive because it considers the social fabric where voices are marginalized or silenced. Radical listeners attend to the social power dynamics between groups (Brekke, 2021). This attention means gaining perspective from “different locales in the social web of reality” (Moore, 2018, p. 481). Thus, listening is an action allowing strategic pause, stillness, and taking stock of socio-political historical locations (Krueger-Henney, 2016). Radical listening also means acknowledging the patterns of “distracted disengagement” that are barriers to listening (Brekke, 2021, p. 96) and shaped by power. One barrier is discussed as “noise,” where dominant narratives and individual beliefs preclude hearing someone (Kress & Frazier-Booth, 2016). For example, dominant narratives about a groups’ (il)legitimacy can be loud when listening. Power dynamics also create barriers to listening via the landscape for dialogue—not everyone is on symmetrical ground for being taken seriously.

Radical listening as a politically engaged form of relationality is important considering adultism is a system of power designed to prime subjects for the normalization of other forms of oppression (Dejong & Love, 2015). In the case of adults and youth enmeshed in adultist social fabric, radical listening implies an anti-adultist standpoint, as well as a way to reflect on, identify, and actively work toward alternative epistemically just relations between teacher and learner. The importance and practice of radical listening outside the classroom to disrupt adultism is our extension.

## Current Study

Critical pedagogy literature, particularly from Kincheloe’s work, has progressively forged a radical love and critical dialogue framework over 20 years. Yet, for non-teacher adults interested in youth collaboration, there are specific areas to theorize and investigate further for theory applicability—like adultism from youth perspectives. To date, few studies have asked youth about their desires from adult collaborators, and fewer draw attention to listening. Using surveys and focus groups, our study investigates the following: (1) what can radical listening offer for youth-adult collaboration, (2) from youth perspectives, what are their experiences with adultism, and (3) what do youth desire from adults in collaboration across sectors?

## Method

### Context

Data collection occurred in a coastal central California county from Fall 2021 through Fall 2022, when schools returned to in-person instruction from remote schooling due to COVID-19 safety guidelines. A sense of community and communal activity engagement was in transition from an isolating time. Data collection occurred right after a school stabbing at a local high school. After George Floyd’s murder by police and the 2020 racial justice uprisings, the school board removed school resource officers from campuses. After the stabbing, tensions at school board meetings rose, and the school board reinstated school resource officers, paired with mental health counselors. Youth were not meaningfully engaged in this decision and in fact, the county has few structures for youth decision-making in civic life. In contrast, surrounding communities incorporate youth in decision-making via structures, including youth commissions.

This study occurred during a transition in county-wide programmatic focus led by a local United Way program. Youth programming shifted to position youth as leaders, focusing on action. The context of post-remote learning, amidst the school stabbing, and emerging spaces for youth civic engagement describe the county-wide circumstances for which youth civic participation was (not) valued. We understand youth perspectives about their participation in communal matters against the backdrop of county-wide practices working toward normalizing youth participation.

### Data Collection

#### Survey

In late 2021, the United Way chapter conducted a youth and adult countywide survey to understand youth community-based experiences, how adults support them, barriers to youth participation, and how youth meaningfully participate in and/or lead community activities. The surveys were available online for completion between November 18, 2021 and January 28, 2022, in English and Spanish. One hundred four youth and 41 adults completed online questionnaires. United Way and the third author worked to design, field, and report the findings. United Way anonymized responses which were shared with the third author after receiving ethics review approval (#HS-FY2022-173). This author summarized survey results in a technical report for United Way, which helped shape their upcoming youth programming.

Surveys included constructs measured with validated scales, including 5 to 7 questions per scale. The youth survey included the following scales: *community values youth* (Syvertsen, et al., 2019), *youth voice in decision making* (Zeldin et al., 2014), *policy control* (Lardier et al., 2018), and questions about if *youth feel embedded in social support networks* (California School Climate, Health, and Learning Surveys, n.d.). The adult survey included scales related to *adult support for youth* (California School Climate, Health, and Learning Surveys, n.d.) and *adult perceptions of barriers to youth participation* (Lekies et al., 2009). We recognized a need for more information about youth-adult partnerships, which led to the focus groups, the primary data for this article.

#### Focus Groups

We conducted focus groups to gather detailed accounts of youth experiences while maintaining breadth across perspectives (D. Morgan, 1997). Through youth advocacy and after-school program networks, United Way recruited eighteen youth participants from three after-school programs, two focused on Latinx well-being and health promotion, and one on youth empowerment. Youth participants were involved in programs where adults and youth were discussing community matters, which is a limitation and advantage. Specifically, youth engaged in out-of-school time programs were recruited, which limits generalizability. Yet, these youth spoke concretely about their perspectives on adult-youth collaboration, which is valuable for understanding the nuances of adult support.

Two questions guided the four focus groups: (1) “what do you think are the important problems in your community” and (2) “what do youth want for adult allyship?” Although this paper focuses on requests for adult accompliceship, we include data from the community problem focus groups because of its utility in identifying accompliceship needs.

Focus groups were designed, facilitated, and transcribed by a research team consisting of three undergraduate femmes of color, and one graduate Chicano (two are paper authors). Three focus groups were held over zoom to provide more accessibility.

Focus groups with youth participants required special consideration for rapport building and flexibility (Dreby, 2015). Facilitators utilized icebreaker activities to initiate conversations. Activities included the river of life (Carmody, 2023) and an open-ended prompt: “Imagine a society where all young people were involved in decision-making and were supported and encouraged by adults around them. What does that society look like? What do we need to get there” (Khanna & McCart, 2007, p. 1)? Afterward, facilitators utilized a semi-structured protocol to elicit responses from youth, flexibly moving the conversations. Focus groups were audio recorded then transcribed for analysis. Zoom chats were saved for analysis.

### Qualitative Data Analysis

Transcriptions of audio and zoom chat responses were analyzed by a similar but different team (two tenured white women, two graduate students [one femme of color, and one Chicano]) for overarching themes. Because we were interested in how youth experienced adultism and their desires for adult support, we utilized a combination of inductive and deductive analysis: reflexive thematic analysis (Braun & Clarke, 2021; Terry & Hayfield, 2021). This technique allowed inductive (theorizing and analyzing from the youth’s requests for support) and deductive (assessing examples aligned with radical listening theories) analysis with a reflexivity process.

The recursive and reflexive analytic process acknowledging the researchers’ values and positionalities (as adults) is fitting considering our research question encompasses listening and allyship. During early analytic phases (familiarization, coding, generating initial themes, reviewing, and refining themes; Brane & Clarke, 2021), we wrote reflexive memos about our positions and perspectives to identify the “noise” getting in the way of hearing and learning (Kress & Frazier-Booth, 2016). Our subjectivities, as adult researchers with years of youth partnership and research experience, were analytic resources (Braun & Clarke, 2021).

During preliminary analyses, the first author checked the credibility of early theme development with two local youth leaders; they affirmed and highlighted the relational themes (Lincoln & Guba, 1985).

## Discernments and Discussion

### Survey

Table 1 aligns survey results with framing concepts we used to analyze the focus groups. Youth reported that although their voices were welcome, their opinions were not taken seriously (*t*[94] = 3.78, *p* < .001, Cohen’s *d* = 0.76). Notably, adults felt more strongly their programs supported youth than was reported by youth (*t*[132] = 2.96, *p* < .01, Cohen’s *d* = 0.63), and adults were generally unaware or unsure of youth barriers to program participation. Although majorities of youth felt supported and heard, sizable minorities did not. We analyzed these data by region of the county, which aligns with county demographics; north county is wealthier and populated more heavily by white families whereas south county includes more working class/poor and Latinx families, including mixed documentation status and/or undocumented families. The majority of youth surveyed wanted their voices to be taken seriously, but youth in north county were more likely to think their voices were taken seriously than youth in south county (*t*[77] = 3.23, *p* < .01, Cohen’s *d* = 1.03). This result mirrors other findings that White youth have more positive perceptions of community programs than Black youth (Jones & Perkins, 2006). In addition, youth appeared to feel more supported by people they knew than valued by the community at large (*t*[94] = 8.07, *p* < .001, Cohen’s *d* = 0.78).

**Table 1.** Overlay of Survey Findings with Radical Listening and Youth-Adult Partnership Frameworks.

Framing concept	Youth survey evidence	Adult survey evidence
Adult support for youth	Supportive adult relationships *R* = 1–5; *M* = 4.12; *SD* = 0.91 Std *M*^1^ = 3.29; *SD* = 0.73	Adults thought youth with whom they worked were well supported in their program *R* = 1–4; *M* = 3.67; *SD* = 0.44
Youth voice	Youth voices mattered in terms of decision making in their program *M* = 3.82; *SD* = 1.05 Youth thought their participation made a difference in their school or community *M* = 3.74; *SD* = 0.94	
Community adults value youth	Youth felt valued by adults around them *M* = 3.50; *SD* = 0.84	Adults perceived little to no barriers to youth participation in their programs *M* = 3.19; *SD* = 0.63

Insights from the survey point to differential perceptions about the support youth receive. Survey results are enriched by the focus groups—specifically, how youth make sense of adultism as an epistemic and relational matter, their desires for adult support, and how relational counseling may help explain the discrepancies between adults and youth perspectives.

### Focus Groups

We generated three overarching themes: epistemic (in)justice (Siry et al., 2016), relationality (Agnello, 2016; Moore, 2018), and counsel, which is not discussed in the radical listening literature. Within these themes, youth discussed how they experience adultism and their requests for accompliceship related to radical listening praxis.

#### Epistemic Injustice: Adultist Narratives Discredit Youth Knowledge and Divide Youth from Adults

Several youth described experiencing adultism along epistemic and relational domains—where their social group as youth was related to their perceived lack of authority, thus separating them from credible adult knowers. For example, several young people described adults perpetuating testimonial injustice by invalidating their capacity as knowers based on age, noting how adults “dismiss” and “belittle” their struggles and how youth community concerns would be “put into view” if they were voiced by adults. Lolita spoke about the age-credibility connection,

There’s some certain adults who we probably experienced who probably think like . . . ‘I’m right, you’re wrong, okay, you’re young, I’m older, like, I’ve experienced way more than you have.’

In these cases, youth described their experience of being insecure knowers—inherently less “correct” than adults. Youth knowledge and perspectives invalidation contributes to bifurcation where age-based groups are considered valid (adults) and “invalid” (youth) knowers (Bell, 1995; Cummings et al., 2023; Dejong & Love, 2015).

When the thoughts, emotions, and perspectives of youth are rendered less valuable within the community, age-based fixed hierarchies are reinforced. Thus, relationships between youth and adults are stunted (Dejong & Love, 2015; Moane, 2011). For example, Nicky demonstrated their awareness of this division related to dominant narratives about responsibility,

I think that has somewhat do with how there’s not the massive relationship of trust between the adults in kids. A lot of adults still think that the children are like irresponsible.

Nicky explained adults do not trust “kids” because they have not proven themselves as capable or reliable. Social constructions about youth and their development as linear shapes the limits of trustworthiness (Kohfeldt & Langhout, 2012; Rogoff, 2003; Taft, 2015). Adultist divisions like these suppress solidarity and relationality across age groups. Yet, youth talked about how relationality can challenge age-based power dynamics.

#### Relationality: A Challenge to Adultism

Consistent with the literature, youth discussed the challenges in their relationships with adults and their desires to foster relationships with adult accomplices who listen to youth perspectives and desires (Fielding, 2001; Zeldin et al., 2013),

I don’t think there’s much we can do because we’re just like [. . .] kids. Like no, no one is going to listen to a bunch of kids and change the whole community just based on like what we’re saying. So I think people like you and what you’re doing is good because they’re giving a voice, on like what we want.

Luna explained how age-based limitations in credibility restricts their ability to work toward community-based justice. Youth desires and potential are often disregarded by adults, blocking dialogic relationships (Hall, 2020). Luna mentioned the focus group facilitation by adults, a dynamic where adults were asking questions and listening with intentions of learning from rather than responding to youth, as an example of the form of listening they might desire. Yet, Luna also said “giving a voice,” which implies an uneven power dynamic because this conceptualization relies on one group (adults) “giving” voice to another group (youth).

Other youth, however, described more relational connections with adults. Araceli, for example, mentioned a more horizontal relationship when suggesting adults and youth attend a “listening circle” as part of a hypothetical curriculum for adult training on collaborating with youth. Listening circles, common in restorative educational practices, are horizontal dialogue formats aiming to redistribute power dynamics for listening/speaking equity (Brown, 2017). Similarly, when asked about what youth want from adults, Lluvia requested a mutual relationship with adults to be fostered,

Maybe just that the mutual respect that we want is so that we, that there’s no intimidation to ask for help when we need it because I’m less likely to ask for help if I think you’re going to belittle me or not create space for me to explain myself fully, so that’s why we want respect.

Lluvia’s request for respect from adults speaks to how working toward a relationality ethic challenges adultism by including listening without judgment, which aligns with radical listening (Winchell et al., 2016).

Youth discussed how relationality built from respect and listening can create space for possibility. When asked for contributions to a hypothetical curriculum to help adults learn how to support young people, Lupita urged adult accomplices to ask youth the specific ways adults can support them, noting “many times they just assume what we want, or what we need, and they don’t really ask what we need.” The listening youth described involved withholding assumptions—likely informed by adultist tendencies—to hear and understand youth more deeply. We interpret Lupita’s response as a glimmer of how listening can shape space for the possible (Hooks, 2003). Yet, listening alone is not enough.

#### Counsel

Youth narratives confirm and connect the literatures on (1) adultism, (2) youth adult partnerships and (3) radical listening—revealing these youth wanted relational listening and epistemic credibility. Missing from the radical listening literature is counsel. How can adults radically listen *and* mentor youth while challenging adultism?

Although less common, youth were quick to point out what counsel looked like when it enacted adultism. For example, Lluvia described how a well-meaning counselor pressured her to seek white-dominated classes to serve as a role model to represent her marginalized community:

I don’t want to be, like, a marker for everybody else if that makes sense. So it can just be a lot to put that on me and be like okay now you have to try even harder because if you don’t reach your goals then these people won’t think THEY’LL be able to.

Although the counselor offered aspirational guidance, Lluvia described not being seen in her full personhood. Lluvia expressed the pressure to be the model for her “people” was unfair and overshadowed her desires.

Counseling and listening to young people from a radical listening perspective means seeing their desires, especially within an adultist, racialized, gendered, and classist context. Lluvia explained how adults have been more radical counselors to youth:

I’m in this committee at my school and I’ve been having a really good time. But there was a time when I didn’t feel supported by the administrators of that committee and so I told some other adults about it and they gave me tools to express how I felt –like I needed more support or I needed to be listened to more. And uh, it was really was important to me because I felt that I felt that- I felt that more prepared to-um challenge what I thought was going wrong.

Lluvia described what could happen when youth have confidence to seek counsel from adults. In this established relationship, youth were believed and seen. Lluvia was able to seek help, and requested resources to challenge adultism themselves. This example demonstrates an important part of radical listening that challenges adultism: adults can stand alongside youth in disrupting adultism.

### Limitations

Although survey data were collected from youth and adults county-wide, focus groups recruited from youth involved in an out-of-school time program. We may have discerned different themes from youth who were not part of a program. For instance, focus group youth had frameworks for understanding power dynamics between youth and adults, as evidenced by their involvement in after-school programs interested in fostering youth leadership. Therefore, their perspectives on adultism focused on aspects of epistemic justice involving testimonial injustice more so than hermeneutical injustice. Conducting focus groups with youth with varying degrees of accessibility to the epistemic resources required to understand adultism may offer additional insights regarding how radical listening may address aspects of hermeneutical injustice. Future research should therefore include youth not involved in a formal program, including youth who have left programs.

Although youth in the focus groups suggested approaches from adults that could embody radical listening like “listen and give more time” and “guide without taking over,” specific tools were not present in these data. Future studies exploring context-specific and successful radical listening tools will be useful for educators, youth advocates, and policy makers.

We did not conduct focus groups with adults. Doing so would likely raise constraints adults experience based on how many youth they are expected to serve or other institutional contexts making radical listening a challenge. Knowing these constraints may have helped us nuance the results. Future studies should ask adults what gets in the way of their radical listening.

## Conclusion

Together, the survey and focus groups describe the role of radical listening in youth-adult collaboration, how youth experience adultism, and their desires for support from adults. Surveys and focus groups suggest adultist ideologies occlude adults from understanding adultism’s discrediting impacts on youth. The discrepancy in adult and youth perceptions of support for young people is evidence adults overestimate the support young people experience. Similarly, in other studies, adults perceived the relationship between them and youth was more positive (in terms of youth involvement, adult involvement, and youth-adult interactions) than youth perceived (Jones & Perkins, 2006). Focus group narratives elaborate age-based discrimination and justification for dismissal and subjugation. Youth noted how age was the basis for perceived untrustworthiness and discrediting of them, which drove a wedge between youth and adults. These results are consistent with theories about how infantilizing and discrediting narratives about youth separate them from adults (Dejong & Love, 2015; Gordon, 2007). This difference signals to adults that there is much for us to learn from youth about their desired support.

Listening to simply *include* youth voices does not ensure youth are taken seriously. Indeed, radical listening is not a panacea. Youth narratives about their desires to be meaningfully listened to with mutual respect and horizontal-ness align with radical listening characteristics (e.g., relational, dialogic, and power sharing for knowledge building). Yet, structural constraints and power differences between youth and adults cannot be erased by listening only, even if that listening is radical. Typically, adults retain access to specific powerful social networks, funding, and more. These results, however, confirm a prior research where youth across sectors reported feeling support when adults use their knowledge to assist with youth projects (Griffith & Larson, 2016). Similarly, youth who viewed themselves as having a full partnership with adults had higher empowerment outcomes than youth who felt their voices were only heard, or when they were supported by adults without full partnership (Zeldin et al., 2017). Youth desire and benefit from being validated as epistemic partners.

Youth narratives describe a textured form of support and counsel aligned with radical listening values of solidarity. Lluvia shared an undesirable form of counsel, flattening their personhood to their ethnic status. Similarly, youth-adult collaboration is problematic when the goal is to mentor youth (L. Camino, 2005). Dominant mentorship forms can naturalize a hierarchical and paternalistic relationship youth in these focus groups did not desire. Instead, youth desired adults to support them in challenging adultism and enacting horizontal collaboration.

Regarding the multi-sector nature of solidarity, the survey results indicated youth were supported by individuals they knew, but less so by the community at large. The focus group only provided vague desires for accompliceship/solidarity from the broader community, even after being explicitly asked about concrete practices in the “imagine a society” question. Because focus groups participants were recruited through organizations, and participated with their peers from that organization, they may have grounded their responses in actual relationships. Potential youth not connected to an organization and participating with unfamiliar peers might offer different narratives about adults at large. Also, support at the broader community level regarding forms of accompliceship/solidarity might be captured through other methods, like youth participatory action research, where youth and adults research and explore examples from other communities and societies, or by asking different questions. For example, because we were interested in solidarity across sectors, both formal and informal, we did not ask what youth wanted from adults in various sectors. This line of questioning may have sparked important conversations about role constraints around formal sectors, such as mandated reporting and other ethical considerations for specific adult roles, such as teachers and coaches.

Youth-adult collaboration models can implement radical listening as praxis and a dynamic commitment to youth desires for allyship/partnership/coalescing. This study, like Jones and Perkins (2006), suggests adults be trained on how to work alongside youth, with youth experiences as a vital foundation.

## Notes

1. In order to compare youth and adult responses, the youth responses needed to be standardized to be on a scale of 1 to 4 rather than 1 to 5.
